# The Influence of Variation in Hexanucleotide Repeat Number in the 5’ Untranslated Region of Cytochrome C Oxidase Subunit 7A1 on the Development of Cognitive Impairment

**DOI:** 10.1002/alz70855_103467

**Published:** 2025-12-24

**Authors:** Joseph Pleen, Katelyn Cunningham, Ryan Goodson, Graham Blackwood, Heather M Wilkins, Russell H Swerdlow

**Affiliations:** ^1^ University of Kansas Alzheimer's Disease Research Center, Fairway, KS, USA; ^2^ University of Kansas School of Medicine, Kansas City, KS, USA; ^3^ University of Kansas Medical Center, Kansas City, KS, USA; ^4^ University of Kansas Alzheimer's Disease Research Center, Kansas City, KS, USA; ^5^ Department of Biochemistry and Molecular Biology, University of Kansas Medical Center, Kansas City, KS, USA

## Abstract

**Background:**

Cognitive decline is a feature seen with aging and is accelerated in Alzheimer's disease (AD). Mitochondrial dysfunction is also a feature of both aging and AD. A previous study identified a common hexanucleotide (AGCCCC) variant in the 5’ untranslated (5’ UTR) region of the Cytochrome C Oxidase Subunit 7A1 (COX7A1) gene through Sanger sequencing in a region of the genome not amenable to short read sequencing. The 5' UTR is upstream of the promoter and is known to impact translational regulation *in vitro*. In order to assess if this variation has an impact on cognition *in vivo* in humans, a cross‐section of older individuals at risk for decline was sequenced.

**Method:**

Nuclear DNA samples were obtained from a cross‐sectional cohort (*N* = 296) of individuals aged 65‐85 from the National Centralized Repository for Alzheimer's Disease and Related Dementias (NCRAD). Quantitative polymerase chain reaction (qPCR) was performed on the 5’ UTR of COX7A1. Sanger sequencing was performed on a subset of samples to confirm variant length. Fragment analysis was performed on all samples and analyzed in GeneMapper to categorize the number of UTR repeats on each chromosome. Cognitive (MoCA z‐score) and demographic (sex, age, education, and APOE status) data were obtained from corresponding participants from the National Alzheimer's Coordinating Center (NACC). Analysis of variance (ANOVA) was performed. APOE e2 carriers were excluded from analysis. The dependent variable was the Montreal Cognitive Assessment z‐score (MoCA z‐score) and independent variables were APOE status (APOE e4 carrier vs non‐carrier) and dichotomized COX7A1 UTR hexanucleotide repeat number (high repeat number and low repeat number).

**Result:**

Both APOE carrier status and COX7A1 UTR hexanucleotide repeat number were found to result in a statistically significant difference in MoCA z‐score.

**Conclusion:**

In a small cross‐sectional cohort (*N* = 296) aged 65‐85 increased hexanucleotide repeat number in the 5' UTR of COX7A1 appears to negatively impact global cognition (MoCA z‐score). Larger, prospective follow‐up studies paired with neurodegenerative biomarkers will need to be performed to assess if this association is secondary to aging, AD neuropathological change, or other associated pathology.

